# The mode of delivery affects the diversity and colonization pattern of the gut microbiota during the first year of infants' life: a systematic review

**DOI:** 10.1186/s12876-016-0498-0

**Published:** 2016-07-30

**Authors:** Erigene Rutayisire, Kun Huang, Yehao Liu, Fangbiao Tao

**Affiliations:** 1 Department of Maternal, Child and Adolescent Health, School of Public Health, Anhui Medical University, Meishan Road 81, Hefei City, Anhui Province 230032 People’s Republic of China; 2 Anhui Provincial Key Laboratory of Population Health & Aristogenics, Hefei City, Anhui Province People’s Republic of China; 3 Department of Public Health Inspection and Quarantine Science, School of Public Health, Anhui Medical University, Hefei City, Anhui Province People’s Republic of China

**Keywords:** Gut microbiota, Bifidobacterium, Bacteroides, Firmicutes, Clostridium, delivery mode, Caesarean section, vaginal delivery, Infants

## Abstract

**Background:**

The human gut is the habitat for diverse and dynamic microbial ecosystem. The human microbiota plays a critical role in functions that sustain health and is a positive asset in host defenses. Establishment of the human intestinal microbiota during infancy may be influenced by multiple factors including delivery mode. Present review compiles existing evidences on the effect of delivery mode on the diversity and colonization pattern of infants gut microbiota.

**Methods:**

Two investigators searched for relevant scientific publications from four databases (Pubmed, Medline, Embase, and Web of Science). The last search was performed on September 21, 2015, using key terms ((delivery mode OR caesarean delivery OR cesarean section OR vaginal delivery) AND (gut microbiota OR gut microbiome OR gut microflora OR intestinal microflora OR microbial diversity) AND (infants OR children)). All included studies described at least two types of gut microbiota in relation to delivery mode (caesarean section vs vaginal delivery) and used fecal samples to detect gut microbiota.

**Results:**

Seven out of 652 retrieved studies met inclusion criteria, were included in systematic analysis. Caesarean Section (CS) was associated with both lower abundance and diversity of the phyala Actinobacteria and Bacteroidetes, and higher abundance and diversity of the phylum Firmicute from birth to 3 months of life. At the colonization level, *Bifidobacterium*, and *Bacteroides* genera seems to be significantly more frequent in vaginally delivered infants compared with CS delivered. These infants were more colonized by the *Clostridium*, and *Lactobacillus* genera. From the reports, it is tempting to say that delivery mode has less effect on colonization and diversity of *Bifidobacteria*, *Bacteroides, Clostridium,* and *Lactobacillus* genera from the age of 6 to 12 months of life.

**Conclusion:**

The diversity and colonization pattern of the gut microbiota were significantly associated to the mode of delivery during the first three months of life, however the observed significant differences disappears after 6 months of infants life. The healthy gut microbiota is considered to promote development and maturation of the immune system while abnormal gut is considered as the major cause of severe gastrointestinal infections during the infancy. Further studies should investigate the diversity and colonization levels of infant gut microbiota in relation to the mode of delivery and its broad impact on infants’ health at each stage of life.

## Background

The term microbiota refers to the sum of all microbial communities living in or on the human body. The human gut mainly the large intestine harbors the greatest numbers of microbiota in the body when compared to other human body niches such as the skin, vagina, mouth, and ears [1, 2]. The human gut contains 10^14^ bacteria which represent 10 times the total number of human cells. Particularly, after bacterial colonization in infant, intestinal microbial composition is unique for each individual, although more than 95 % can be assigned to four major phyla: Firmicutes, Bacteroidetes, Actinobacteria and Protecteobacteria [3]. The development of the intestinal microflora starts at birth, several reports highlighted that the early life development of the infant gut microbiota plays an important role in the maturation of the host immune system, protection from pathogens, and in providing nutrients [4, 5]. The main function of intestinal immune system is to control the exposure of bacteria to host tissues and prevent the potential for pathologic outcome. It has been suggested that innate and adaptive immune responses influence enteric bacteria composition and luminal alterations of decreased bacterial diversity and expansion of selected species during inflammation of various causes [6]. According to the review, the immune system on microbiota may evolve in immune deficiencies that alter microbial communities in ways that predispose the host to diseases [7]. Specifically, postnatal gut function and immune development are largely influenced by the intestinal microbiota, emerging evidence has shown that early microbiota colonization may influence the occurrence of later diseases [8]. In human, West et al. [6] observed that low microbial diversity in infancy precede onset of allergic disease. Specifically, Azad et al. [9] found more abundant *enterobacteriaceae* and less abundant *bacteroidaceae* in the gut microbiota of food sensitized infants at 3 months and 1 year. The diversity and colonization pattern of infants gut microbiota during first year of life are influenced by numerous factors, of which delivery mode is an important one. Furthermore, evidence is growing that an aberrant gut microbiota composition as a result of delivery mode affects the subsequent regulation of immune response [10], and the changes in the composition of the gut microbiota (dysbiosis) may be associated with several clinical conditions [11]. Recent studies reported increased risk of asthma [12], obesity [13], celiac disease [14], and type 1diabetes [14] in children born via CS compared with vaginally delivered. The possible explanation for this increased risk is that the lack of contact at birth with maternal vaginal and intestinal flora would expose these children to a number of diseases because of changes in the development of immune system [15]. Furthermore, independently to the mode of delivery, low total diversity of the gut microbiota during the first year of life was reported to be associated with allergic diseases [16]. Modulation of gut microbiota with probiotics, prebiotics, or fermented dairy products has been suggested as a treatment of, or prevention for, different disorders such as irritable bowel syndrome (IBS), infectious diarrhea, allergic disease, and necrotizing enterocolitis [8].

The gut microbiota colonization patterns of infants’ delivery by Caesarean Section (CS) differ from those who were delivered vaginally [17]. In the last two decades CS has been on the rise worldwide [18]. At the same time infants delivered by CS were found to have abnormal gut microbiota [19–21]. Considering the pattern of infants gut microbiota, previous studies reported delay or absence of *Bacteroides* in the first year of life in infants delivered by CS [20, 22]. In contrast, infant gut bacterial dominated by clostridial was found not associated with delivery mode [23]. A study conducted in developing country reported early colonization of *Bacteroides* in infants delivered by CS; this was attributed to the fecal bacteria in surrounding environment. For instance phylum diversity within Actinobacteria was higher among vaginally delivered (VD) infants [24]. However, the relation between those patterns with delivery mode remains unclear. Conflicting results observed in several previous studies may not all be attributed to techniques used to detect fecal microbiota in early infancy, as anaerobic genera or facultative anaerobes could be detected by both culture-dependent methods and culture-independent techniques.

In addition to delivery mode, other factors such as place of birth, maternal vaginal or skin microbiota, type of infant feedings, birth weight, gestational age at birth, hospitalization after birth, prenatal administration of probiotics, and intrapartum antibiotics prophylaxis were found to influence the pattern of infant gut microbiota [25]. However, the implication of some factors on the abundance of some specific gut bacterial was refuted in some studies [19, 23, 26, 27]. In facts, detailed discussion on these factors is not in the scope of this systematic review. So far there is insufficient explanation regarding the pattern and diversity of infant gut microbiota in relation to delivery mode. To better elucidate this relation, the present systematic review intends to look whether the mode of delivery affect the diversity and microbial colonization pattern during the first year of infants' lives.

## Methods

### Eligibility criteria

#### Inclusion

Included in this systematic review are studies with human subjects including newborns delivered either vaginally or by CS. The following clinical data must be considered in the study: birth weight, gestational age, types of infant feeding, and antibiotics use. Included studies relate pattern colonization of infant gut microbiota with delivery mode and reported the colonization rate of gut microbiota over time. Birth cohort studies using fecal stool sample to detect gut microbiota were included in the systematic review.

#### Exclusion

**Fig. 1:**
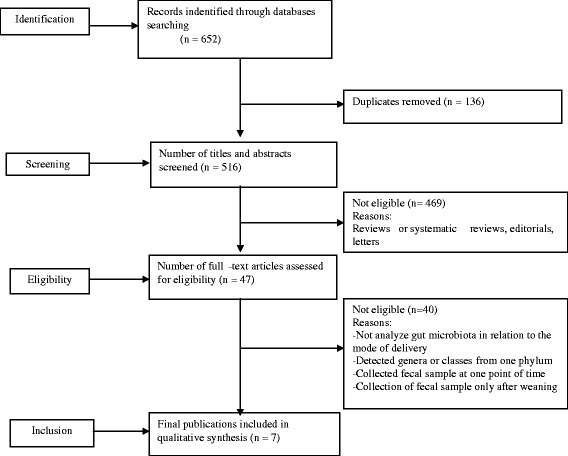
A flow chat diagram of screening and selection processes

Studies which reported the collection of fecal sample only after weaning were excluded [28, 29]; these studies lack the fecal sample in the first days after birth. Given that, the progressive or alteration of gut microbiota could not be explained. Studies which collected fecal sample at one point of time [19, 30], detected genera or classes from one phylum [31] and drawn conclusion based on gut microbiota detected in the subjects from different countries [32] were excluded. One cross-sectional study was excluded because of small sample in VD infants, and did not contains analysis of longitudinal changes in the intestinal microbiota over time [33]. Several studies were excluded because they didn’t report infants’ gut microbiota in relation to the mode of delivery, and not use fecal sample to detect gut microbiota (Fig. 1).

### Study selection

After the search terms and electronic database were agreed upon, two authors were independently assessed titles and abstracts to determine eligible studies. Disagreements between the two authors were resolved by mutual agreement. If not, a third author intervened for the final decision.

### Information sources and searching strategy

We searched four electronic databases (PubMed, Medline, Embase and Web of Science) using different key terms such as (delivery mode OR caesarean delivery OR cesarean section OR vaginal delivery) AND (gut microbiota OR gut microbiome OR intestinal bacterial OR gut microflora OR microbial diversity) AND (infants or children). Also, to supplement these search results, we searched reference lists of the reviewed studies. To be included in the systematic review, studies had to be: published in English language journal, and published before September 21, 2015. Studies with abstract or full text were considered during the records identification. Non- English language articles were screened, and assessed for inclusion.

### Data collection process and list of item

Data collection was independently completed by two authors by using predefined data extraction form and crosscheck was performed to make sure that the eligible studies met inclusion criteria. In case a study tested fecal sample of mother-infant pairs and showed the structure of gut microbiota in fecal samples separately, only the infants gut microbiota were considered.

The following data were extracted from the study selected for review: (1) publication year and country of origin, (2) periods of sample collections, (3) gestational age, (4) number of infants by delivery mode (VD vs CS), and (5) bacterial detection techniques.

The methodological quality of the studies was assessed by examining subjects selection, sample size, loss to follow-up, techniques used to determine infants gut microbiota, and inclusion of potential confounding variables such as infants feeding types, birth weight, gestational age, antibiotics use and health status.

### Data analysis

The gut microbiota were grouped according to detection periods, from birth to 7 days, 8–30 days, 31–90 days, 91–180 days and 180–360 days and then analyzed prior to delivery mode. Studies with the same bacterial species were analyzed together according to the period of fecal sample collection. Significant difference observed for specific gut microbiota of phyla diversity was reported at each point of time for all studies. Individual colonization pattern of gut microbiota was considered during analysis where applicable.

## Results

### Study selection

The search of electronic databases and additional search from reference list yielded to 652 studies, 136 were directly excluded after duplicate removal, and 469 records were excluded after screening of abstracts and titles. Excluded studies were reviews, animal studies or studies not relate delivery mode to the microbial colonization pattern or phyla diversity of infant gut microbiota. The full texts of the remaining 47 studies were deeply assessed for inclusion, 40 studies did not meet all our inclusion criteria (Fig. 1). Finally seven studies were included in the present systematic review.

### Study characteristics

Our search strategies provided seven studies which met all the inclusion criteria, used fecal sample to detect infants gut microbiota pattern at different points of time during early infancy. Apart from one study [34], other studies reported small number of infants in CS group [20, 21, 26, 27, 35, 36] . All studies compared at least two types of gut microbiota from different phyla with the mode of delivery. Included studies are arranged according to publication year (Table 1). Detailed information on infants’ feeding by delivery mode were available in two studies [20, 34], and not in other five studies [21, 26, 27, 35, 36]. Regardless of the mode of delivery, very few infants had exclusively breast-fed for at least six months of age. In all the seven included studies, the majority of mothers who gave birth through CS were given antibiotics few hours before operation. No infant received antibiotics during the neonatal period in all included studies.

**Table 1:** Characteristics of included studies

Author	County	Gestation period (Mean, wk)	Publication year	VD	CS	Period for sample collection	Microbial isolation and analysis techniques
[35]	Singapore	38.87	2015	57	18	3d, 3wk, 3Mo, and 6Mo	DNA extraction and 16S rRNA gene amplification
[36]	India	36	2013	73	10	1d, 2d, 4d, 7d, 14d, 28d, 90d, and 180d	DNA extraction and 16S rRNA gene sequencing
[27]	Sweden	–	2013	15	9	1wk, 1Mo, 3Mo, 6Mo, 12Mo and 24Mo	DNA extraction and 16S rRNA gene amplification
[34]	Greek	39.05	2008	34	48	4d, 30d and 90d	Culture-dependent and molecular methods.
[21]	Finland	39.35	2008	141	24	1Mo, 3Mo,6Mo, and 12 Mo	Fluorescence in situ hybridization of bacterial cells.
[20]	Finland	39.5	1999	34	30	3d,5d,10d,30d,60,180d	Cultured on nonselective and selective media
[26]	Sweden	–	2014	109	19	6d,3wk,2Mo,6Mo	DNA extraction and 16S rRNA gene sequencing

d-day, wk-week, Mo-month

### Diversity and Bacterial colonization pattern in the first year of life

#### Microbial diversity and colonization pattern from birth to 7 days of life

Low total diversity of the gut microbioata during the first week of life was reported in the infants delivered by CS. In four studies, phylum diversity within Actinobacteria such as *Bifidobacterium* was significantly lower in the infants delivered by CS compared to vaginally delivered infants [20, 26, 34, 35]. In contrast, one study found no significant difference regarding the abundance of *Bifidobacterium* among the infants delivered by different modes [36]. Within Proteobacteria phylum, the infants delivered by CS had a lower abundance of *Enterobacteriaceae* compared with those delivered vaginally [36]. Dissimilarly, significantly higher abundance of unclassified *Enterobacteriaceae* among infants delivered by CS was reported in one study [26].

Total diversity of the gut microbiota from Bacteriodetes phylum was lower among infants delivered by CS. Four studies consistently reported lower colonization pattern of the *Bacteroides* in infants delivered by CS compared with the vaginally delivered at the age of 1 day [36], at the age of 3 days [20], at the age of 6 days [26], and at the age of 1 week [27].

In two studies, analysis to the phylum level showed no significant differences within the Firmicutes phylum between CS and vaginally delivered infants [27, 35]. Specifically, the colonization levels of several genera belonging to Firmicutes phylum, for example *Clostridium perfringens*, *Enterococcus-like*, *Lactobacillus-like* bacteria were not significantly influenced by the mode of delivery [20, 34]. However, the genus of *Lactobacilli* was significantly more frequent in vaginally delivered than CS delivered infants [34, 36]. Significantly higher abundance of *Haemophilus*, *Veillonella*, *Clostridiaceae1* and *Klebsilla* bacteria were observed in CS delivered infants compared with the vaginally delivered [26, 35]. In summary, infants delivered by CS have lower total gut microbiota diversity in the first week of life compared with vaginally delivered. However, colonization pattern of some genera from Firmicutes phylum are not influenced by the mode of delivery.

#### Infant’s Microbial diversity and colonization pattern from 8 to 30 days of life

Several studies reported the infant’s microbiota diversity and colonization pattern from the age of 8 to 30 days of life. The Actinobacteria (dominated by *Bifidobacterium*) was the dominating phylum in vaginally delivered infants after the first week of life [20, 21, 26, 34, 35]. The colonization rates of *Bifidobacterium* genus was statistically lower in the CS delivered than in the vaginally delivered infants at age of 10 days [20], at the age of 30 days [34], at the age of 1 month [21], and at the age of 3 weeks [26, 35]. In contrast, *Bifidobacterium* genus dominated the microbiota in both infants delivered by CS and vaginally, no significant difference between the infants delivered by different mode and colonization of *Bifidobacterium* at the age of 30 days [20], at the age of 28 days [36], and at the age of 1 month [27]. Within Proteobacteria phylum, infants gut microbiota colonization pattern of the *Enterobacteriaceae* genus was not significantly influenced by the mode of delivery at the age of 28 days in one study [36].

Vaginally delivered infants were colonized by *Bacteroides* to a greater extent than CS delivered with significant difference in three studies, at the age of 10 and 30 days [20], at the age of 28 days [36], and at the age of 3 weeks [26] but no significant difference in the number of *Bacteroides* colonization between vaginally delivered infants and those delivered by CS observed in one study at the age of 1 month [21].

In four studies, no significant differences were observed in the gut microbiota dominated by species of *Lactobacillus-like* at 10 and 30 days [20], of *Lactobacillus* at the age of 1 month [21], at the age of 28 days [36], of *Enterococcus-like* at the age of 30 days [34] and of *Clostridium perfringens* at the age of 10 days [20] among the infants delivered by different modes. In contrast, CS delivery was associated with significantly higher abundance of the *Clostridium* genus at the age of 3 weeks [26], and the *Enterococcus* genus at the age of 1 month [27]. In summary, *Bifidobacterium* genus are the dominating gut microbiota in the vaginally delivered infants, while *Clostridium* genus are more abundant in CS delivered. Moreover, CS delivery is associated with significantly lower total microbiota diversity at the age 30 days.

#### Infant’s Microbial diversity and colonization pattern from 31 to 90 days of life

Higher total diversity of the infants gut microbiota dominated by *Bifidobacterium* genus was reported in vaginally delivered infants by two studies, at the age of 90 days [36], and at the age of 3 months [34]. In Contrast, two studies reported no significant association between delivery mode and colonization of *Bifidobacterium*, at the age of 60 days [20], and at the age of two months [26]. Within Proteobacteria phylum, almost similar *Enterobacteriaceae* genus was observed in both infants delivered by CS and vaginally, and the colonization of this genus was not associated with delivery mode, at the age of 90 days [36], and at the age of 2 months [26].

In four studies, delivery mode affects the diversity of the Bacteroidetes phylum, with significant higher levels of the *Bacteroides* at the age of 2 months [26], *Bacteroides* at the age of 3 months [27], *Bacteroides-fragilis* at the age of 2 months [20], and of the *Bacteroides-prevotella* at the age of 90 days [36] in vaginally delivered infants than in CS delivered.

Diversity of the Firmicutes phylum did not differ between the infants delivered by different mode at the age of 3 months [27]. Within this phylum, colonized with the *Lactobacillus-like*, and *Clostridium perfringens* at the age of 60 days [20], *Enterococcus* at the age of 3 months [27] and *Lactobacillus* at the age of 90 days [36] were not significantly associated with the mode of delivery. However, one study reported significantly higher abundance of the *Clostridium* genus in the CS delivered infants compared with the vaginally delivered at the age of 2 months [26]. At the phylum level, the Bacteroidetes diversity dominated by Bacteroides genus are significantly lower in CS delivered infants compared with vaginally delivered at the age of 3 months. Colonized by several genera belonging to Firmicutes phylum are not significantly associated to the mode of delivery.

#### Infant’s Microbial diversity and colonization pattern from 91 to 180 days of life

In five studies, the colonization pattern of the *Bifidobacterium* were not associated with the mode of delivery, at the age of 6 months [21, 26, 35], and at the age of 180 days [20, 36]. Within the phylum of Proteobacteria, colonized by the genus of the *Enterobacteriaceae* was not significantly associated with the mode of delivery at the age of 180 days [36]. In one study, the colonization pattern of the *Bacteroides fragilis* was significantly higher in vaginally infants compared to CS delivered at the age of 6 months [20], however three studies observed no significant differences between the mode of delivery and the colonization pattern of the *Bacteroides* [21, 26, 27, 36]. In addition, no significant difference observed in one study, when compared the colonization pattern of the *Clostridia* in the vaginally versus CS delivered infants at the age of 6 months [21, 26]. Similarly, the colonization pattern of the *Clostridium perfringens* was not associated with the mode of delivery at the age of 180 days [20]. Similarly, in three studies, colonized by the genus of the *Lactobacillus* was not significantly associated with delivery mode at the age of 6 months [20, 21, 36]. The phyla diversity within Actinobacteria, Proteobacteria, Bacteroidetes and Firmicutes at the age of six months of life was not associated with delivery mode. At this age, the infants delivered both by CS and vaginally were colonized almost by the same species.

#### Infant’s Microbial diversity and colonization pattern from 181 to 360 days of life

In one study, the Bacteroidetes diversity was significantly lower in the infants delivered by CS than vaginally delivered, at the same time the diversity of Firmicutes was significantly lower in the CS delivered infants at the age of 12 months [27]. The *Bifidobacterium* was the dominant genus at the age of 12 months but the abundance of this genus was not affected by the mode of delivery. Moreover, no specific genera from any phyla influenced the colonization of CS or vaginally delivered infants at the age of 12 months.

## Discussion

In this review we emphasized on the gut microbiota commonly detected by both culture based method, and culture-independent techniques and this gives us the broad view on the common gut microbiota that colonized infants during their first year of life with respect to the mode of delivery. From birth to 90 days of life, phyla diversity within Actinobacteria and Bacteriodetes such as *Bifidobacterium*, *Bacteroides* were significantly lower in the infants delivered by CS compared with those delivered vaginally. In fact, the mode of delivery affects the colonization pattern of infants’ gut microbiota within these periods but the constituent genera from Firmicutes phylum (dominated by *Clostridium* and *Lactobacilli*) are independently associated with the mode of delivery. The discrepancy within reviewed studies regarding the colonization pattern of the genera of *Clostridia* and *Lactobacilli* in relation to the mode of delivery may due to the microbiota isolation and analysis techniques [20, 36]. The findings of previous cultured-based studies, showed contradicted results, for example Mitsou et al. [34] found significantly lower colonization rates of the *Bifidobacterium* genus at the age of 4 and 30 days, the *Lactobacillus* genus at the age of 4 days in the CS delivered infants compared with vaginally delivered at the age of 30 days while Gronlund et al. [20] could not confirm these associations. In addition, Bezirtzoglou [37] reported the similar colonization of the *Bifidobacterium* genus in both infants delivered by CS and vaginally. However, the author’s conclusion was based on the studies which used culture-based method to detect infants gut microbiota. Contradicted results within these studies may due to other methodological aspects, not necessarily due to microbial isolation and detection techniques. Interestingly, the findings of Gronlund et al. [20] have been collaborated by several more recent culture-independent studies, which reported no significant association between the colonization by the *Clostridium*, and *Lactobacillus* genera and the mode of delivery at the age of one month [21, 36]. In contrast, a birth cohort study conducted in three European countries, confirmed high prevalence of *Clostridia* in CS delivered infants compared with vaginally delivered [38]. Another study conducted in India highlighted the high abundance of *Clostridium difficile* in CS infants and absence of those bacteria in VD infants [30].

Recent studies used modern technology to analyze the composition of the human gut microbiota over time. For instance, a cross-sectional study used polymerase chain reaction (PCR)-denaturing gradient gel electrophoresis in combination with 16r RNA gene sequencing to analyze profiles of the fecal microbiota have reported the total absence of the *Bifidobacterium* genus in infants delivered by CS at the age of 4 days [33]. Biasucci et al. [19] used PCR-denaturing gradient gel electrophoresis (DGGE) and PCR-temperature gradient gel electrophoresis (TGGE) to analyze infants gut microbiota found that total microbial diversity was influenced by the delivery mode, with significantly lower abundance of the *Bifidobacterium* genus at the age of 3 days of life. Indeed, low total diversity of the gut microbiota during the first 3 months of life is associated with Caesarean section. The beneficial health effects attributed to the bacteria communities from the genera *Lactobacillus* and *Bifidobacterium* included the fed as probiotics to promote health and effective in prevention of gastroenteritis in early infancy [39, 40]. Specifically, infants delivered by CS were colonized by high abundance of *Enterococcus*, *Klebsieella*, *streptococcus*, *Haemophilius* and *veillonella* from first week of life to one month [26, 27, 32, 33, 35]. *Veillonellaceae* and *Enterobacteriaceae* bacteria were more abundance in CS infants compared to VD infants [41]. These types of bacteria were not fully studied in relation to the mode of delivery, and explore their benefits to human health and diseases.

Lower abundance of the *Bifidobacteria* and *Bacteroides,* and higher abundance of *Clostridia,* and *lactobacilli* in the infants delivered by CS as observed in some studies, may explained by the antibiotics use. Mothers undergoes CS are more likely to take antibiotics before, during, and after delivery, which may affect the diversity of infants gut microbiota. Previous studies found antibiotic consumption as potential factor that influences the composition of the infant gut microbiota. Specifically, postnatal consumption of antibiotic was associated with higher relative abundance of the *Clostridium leptum*, and decreased numbers of *Bifidobacterium* and *Bacteroides* [32, 42, 43]. The lack of contact with maternal vaginal microbiota, may be a possible reason of higher abundance of genera from Firmicutes phylum and lower colonization pattern of the genera from the Bacteroidetes phylum among CS delivered infants. Previous studies suggested that the genus *Bacteroides* may be transmitted from the maternal gut to new born during delivery [38, 44], while higher abundance of the genus *Clostridium* among CS delivered infants was attributed to nosocomial infection [43].

Caesarean delivery reduces breastfeeding in the first hour of life, which may complicate the management of lactation in the first year of infant’s life. Bai et al. indicated that shorter duration of any breastfeeding was associated with CS delivery [45]. This may disrupt the diversity and colonization pattern of infants gut microbiota. In contrast, world literature meta-analysis on the association between CS and breastfeeding, reported that CS had no significant effect on any breastfeeding at the age of 6 months [46]. However, formula feeding has been associated with higher microbial diversity [47], high abundance of *Clostridium difficile* [48], and lower abundance of *Bifidobacteria* [49]. Indeed, higher abundance of the *Bifidobacterium* genus was observed in breastfeed infants [48]. The findings from a large cohort of Danish infants reported a significant changes in the gut microbiota occurred from age 9 to 18 months, when cessation of breastfeeding and introduction of a complementary feeding induce replacement of a microbiota dominated by *Clostridium* and *Bacteroides* species [50]. It was recently shown that human milk contains beneficial factors for the intestinal microbiota, such as human milk oligosaccharides (HMOs), this function as prebiotics by stimulating the growth of *Bifidobacterium* and *Lactobacillus* species, thereby selectively altering the microbial composition of the intestine [51]. Moreover, there is accumulating evidence that human milk is not sterile but contains maternal derived bacterial molecular motifs that are thought to influence the newborn’s immune system development [52]. It was suggested that host defenses can be improved by feeding breast milk, which helps the immature intestinal mucosal immune system to develop and respond appropriately to highly variable bacterial colonization and food antigen loads [8].

Given the evidence that infants delivered by CS lacked the early support of breast milk as stimulator for a physiological gut microbiota [15], and thus breast milk contains microbes such as *Lactobacilli* and *Bifidobacteria* [53] and this may be a direct source of higher colonization rates of these genera in vaginally delivered than in CS delivered infants. It is plausible that lower total microbial diversity observed from birth to the age of 90 days in CS delivered infants may contribute to weak immune system, poor health and infant well-being. As result of changes of early life gut microbiota, mounting evidences showed that infants delivered by CS had increased risk of developing immune related disorders in childhood, including atopic or allergic diseases [54], inflammatory bowel disease [55], asthma [56], and metabolic disorders [13, 57].

In subsequent days, from the age of 91 to 360 days the effects of the mode of delivery on the diversity and colonization pattern of infant gut microbiota decreased. Within mentioned periods, two studies reported significantly lower abundance of the *Bifidobacterium* and *Bacteroides* genera in CS delivered infants, whereas only one study observed significantly higher abundance of *unclassified Enterobacteriaceae* and *Clostridiales* in CS delivered infants (Table 2). However, no difference was observed between *Bacteroides* colonization and the mode of delivery at the age of 6 months [20], and at the age of 12 months [26]. Similarly, colonized by the genus *Lactobacillus* was not significantly associated to the mode of delivery at the age of 6 months [21]. In contrast, Yap et al. [32] found lower abundance of *Lactobacilli-enterococci* in vaginally delivered infants at the age of 12 months.

**Table 2:** Microbiota colonization pattern significantly associated to the mode of delivery at different postnatal periods in the first year of life

Period for fecal sample collection	Type of Bacteria species identified	Colonization rates		*P*-value	Interpretation	Reference
		Vaginally delivered Infants	CS delivered Infants			
From birth to 7 days	Actinobacteria					
	Bifidobacterium- like bacteria	41 %	4 %	*P* = 0.003	Lower in the CS delivered infants	[34]
	Bifidobacterium OTU1	28 %	6 %	*P* = 0.042	Lower in the CS delivered infants	[35]
	Bifidobacterium – like	85 %	36 %	*P* < 0.001	Lower in the CS delivered infants	[20]
	Bifidobacterium	–	–	*P* < 0.05	Lower in the CS delivered infants	[26]
	Proteobacteria					
	Enterobacteriaceae	–	–	*P* = 0.0174	Lower in the CS delivered infants	[36]
	Unclassified Enterobacteriaceae	–	–	*P* < 0.05	Higher in the CS delivered infants	[26]
	Haemophilus	–	–	*P* < 0.05	Higher in the CS delivered infants	[26]
	Bacteriodetes					
	Bacteroides	73 %	11 %	*P* = 0.005	Lower in the CS delivered infants	[27]
	Bacteroides – prevotella	–	–	*P* = 0.0130	Lower in the CS delivered infants	[36]
	Bacteroides fragilis	68 %	3 %	*P* < 0.001	Lower in the CS delivered infants	[20]
	Bacteroides	–	–	*P* < 0.05	Lower in the CS delivered infants	[26]
	Firmicutes					
	Lactobacilli	59 %	4 %	*P* = 0.000	Lower in the CS delivered infants	[34]
	Lactobacillus	–	–	*P* < 0.001	Lower in the CS delivered infants	[36]
	Clostridiaceae1	–	–	*P* < 0.05	Higher in the CS delivered infants	[26]
	Veillonella			*P* < 0.05	Higher in the CS delivered infants	[26]
	Klebsiella	–	–	*P* = 5.8E^−8^	Higher in the CS infants	[35]
From 8 to 30 days	Actinobacteria					
	Bifidobacterium-like	53 %	0	*P* = 0.007	Not detected in the CS delivered infants	[34]
	Bifidobacterium-like	98 %	58 %	*P* < 0.001	Lower in the CS delivered infants	[20]
	Bifidobacterium	–	–	*P* < 0.05	Lower in the CS delivered infants	[26]
	Bifidobacteria	–	–	*P* = 0.001	Lower in the CS delivered infants	[21]
	Proteobacteria					
	Unclassified Enterobacteriaceae	–	–	*P* < 0.05	Higher in the CS delivered infants	[26]
	Haemophilus	–	–	*P* < 0.05	Higher in the CS delivered infants	[26]
	Bacteroidetes					
	Bacteroides-prevotella	–	–	*P* = 0.0338	Lower in the CS delivered infants	[36]
	Bacteroides fragilis	63 %	0	*P* < 0.001	Not detected in the CS delivered infants	[20]
	Bacteroides	–	–	*P* < 0. 05	Lower in the CS delivered infants	[26]
	Bacteroides	–	–	*P* < 0.05	Lower in the CS delivered infants	[27]
	Firmicutes					
	Clostridium perfringens	17 %	57 %	*P* = 0.003	Higher in the CS delivered infants	[20]
	Clostridium	–	–	*P* < 0.05	Higher in the CS delivered infants	[26]
	Veillonella	–	–	*P* < 0.05	Higher in the CS delivered infants	[26]
	Enterococcus	–	–	*P* < 0.0001	Higher in the CS delivered infants	[27]
From 31 to 90 days	Actinobacteria					
	Bifidobacterium	–	–	*P* = 0.0064	Lower in the CS delivered infants	[36]
	Bifidobacterium	–	33 %	*P* = 0.046	Lower in the CS delivered infants	[35]
	Proteobacteria					
	Unclassified Enterobacteriaceae	–	–	*P* < 0.05	Higher in the CS delivered infants	[26]
	Bacteroidetes					
	Bacteroides-prevotella	–	–	*P* = 0.0336	Lower in the CS delivered infants	[36]
	Bacteroides	73 %	11 %	*P* = 0.005	Lower in the CS delivered infants	[27]
	Bacteroides fragilis	78 %	14 %	*P* < 0.001	Lower in the CS delivered infants	[20]
	Bacteroides	–	–	*P* < 0.05	Lower in the CS delivered infants	[26]
	Firmicutes					
	Clostridium	–	–	*P* < 0.05	Higher in the CS delivered infants	[26]
From 91 to 180 days	Actinobacteria					
	Bifidobacterium	–	40 %	*P* = 0.046	Lower in CS delivered infants	[35]
	Proteobacteria					
	Unclassified Enterobacteriaceae	–	–	*P* < 0.05	Higher in the CS delivered infants	[26]
	Bacteroidetes					
	Bacteroides fragilis	76 %	36 %	*P* = 0.009	Lower in the CS delivered infants	[20]
	Firmicutes					
	Clostridiales	–	–	*P* < 0.05	Higher in the CS delivered infants	[26]
From 181 to 360 days	Bacteroidetes					
	Bacteroides	93 %	44 %	*P* = 0.015	Lower in the CS delivered infants	[27]

“-“data not available

Following 6 months of age, in both culture-dependent and culture-independent studies, no difference observed between the vaginally delivered and CS delivered infants regarding the colonization by the *Bifidobacterium, Bacteroides*, *Clostridium*, and the *Lactobacillus species* [20, 21, 26, 36]. Some of these studies have also revealed that total microbiota diversity not differs between vaginally and CS delivered infants. In contrast, total microbial diversity was found significantly higher in vaginally delivered infants compared to CS delivered [27, 32]. Moreover, we could not attribute the observed differences to methodology used, as some reviewed studies reported almost the similar types of infant gut microbiota independently to bacterial detection techniques. Instead, some studies found small numbers of CS infants colonized by similar genera with their vaginally delivered counterparts. Specifically, the lower colonization of *Bifidobacterium* and *Bacteroidetes*, and high abundance of genera from *the Firmicutes phylum* after the age of 6 months of life may not attributed to Caesarean delivery rather than other environmental factors. The diversity of infants gut microbiota may be influenced by geographical variation such as latitude. We therefore suggested that the conflicting results reported in reviewed studies may due to the demographic factors and structure of infants stool microbiota during the first year of life. As previous reported that population living in different latitudes may differ from *Firmicutes* and *Bacteroides* colonization levels [58, 59]. The major limitation in the present systematic review is that few studies have reported on microbial diversity or colonization level of the gut microbiota in relation to the mode of delivery from 6 to 12 months of infants’ life. Furthermore, the effects of breastfeeding, antibiotic use during or after pregnancy were not mentioned in some reviewed studies.

## Conclusion

CS delivery is associated with a lower abundance and diversity of the phyala Actinobacteria, Bacteroidetes*,* and higher abundance and diversity of the Firmicute phylum at the first 3 months of life but not in the following periods. At the colonization level, *Bifidobacterium*, and *Bacteroides* genera seems to be significantly more frequent in vaginally delivered infants compared with CS delivered, these infants are more colonized by the *Clostridium*, and *Lactobacillus genera* at the first 3 months of age, but not in the subsequent periods. From the reports, it is tempting to say that delivery mode has less effect on colonization of *Bifidobacteria*, and *Bacteroides* at six and 12 month of age. The high abundance of *Bifidobacterium* species in infants is considered to promote development and maturation of the immune system to sustain health and high abundance of C*lostridium difficile* is considered as one of the major nosocomial threats causing severe gastrointestinal infections during the infancy. Future studies should deeply investigate colonization pattern of infant gut microbiota in relation to delivery mode and its broad impact on infant’s health at each stage of life, special attention to the demographics factors should be considered. Also, increasing the sample size in both groups could be considered by future studies.

## Abbreviations

CS, Cesarean Section; PCR, Polymerase Chain Reaction; TGGE, Temperature gradient gel electrophoresis; VD, Vaginal Delivery
